# Dissecting the genetic architecture of *Fusarium verticillioides* seed rot resistance in maize by combining QTL mapping and genome-wide association analysis

**DOI:** 10.1038/srep46446

**Published:** 2017-04-19

**Authors:** Ming Ju, Zijian Zhou, Cong Mu, Xuecai Zhang, Jingyang Gao, Yakun Liang, Jiafa Chen, Yabin Wu, Xiaopeng Li, Shiwei Wang, Jingjing Wen, Luming Yang, Jianyu Wu

**Affiliations:** 1 College of Agronomy, Henan Agricultural University, Zhengzhou 450002, China; 2 Collaborative Innovation Center of Henan Grain Crops, Henan Agricultural University, Zhengzhou 450002, China; 3 College of Life sciences, Henan Agricultural University, Zhengzhou 450002, China; 4 College of Horticulture, Henan Agricultural University, Zhengzhou 450002, China

## Abstract

*Fusarium verticillioides* can be transmitted via seeds and cause systemic infection in maize (*Zea mays* L.); its mycotoxin has harmful effects on animal and human health. We combined QTL mapping in recombinant inbred line (RIL) populations with a genome-wide association study (GWAS) of 217 diverse maize lines using 224,152 single nucleotide polymorphisms (SNPs) under controlled conditions to determine the genetic architecture of *F. verticillioides* seed rot (FSR) resistance. Our study identified 8 quantitative trait loci (QTLs) and 43 genes associated with 57 SNPs that were correlated with FSR resistance through linkage mapping and GWAS, respectively. Among these, there were three candidate genes, namely *GRMZM2G0081223, AC213654.3_FG004*, and *GRMZM2G099255*, which were detected in both linkage mapping and GWAS. Furthermore, the near-isogenic lines (NILs) containing *GRMZM2G0081223*, which also had a susceptible parent background, were found to have a significantly improved level of resistance. In addition, the expression profile of the three candidate genes revealed that they all respond to the infection following inoculation with *F. verticillioides*. These genetic analyses indicate that FSR resistance is controlled by loci with minor effect, and the polymerization breeding of lines with beneficial alleles and candidate genes could improve FSR resistance in maize.

Maize is one of the most important cereals in the world due to its high yield potential and its high demand for use as food, feed, and for industrial purposes. Maize is subject to a variety of biotic and abiotic stresses during its lifetime, which can significantly affect final yield and quality. The planting area for maize in China is 37.07 million hectares, of which the Huang-Huai-Hai region accounts for 39%. In this region, the incidence of soil-borne diseases in maize has increased over the years, which coincides with increases in the use of cultivation systems such as double cropping, no-tillage, and straw returned. *Fusarium verticillioides* (formerly *Fusarium moniliforme*), which is a commonly-found soil-borne fungal species, can be transmitted to seeds and cause systemic infection in maize^1^. Previous studies have confirmed that seeds infected with *F. verticillioides* are a source of root and stalk infection^2^. In addition, the fungus can be transmitted from the planted seed through to the developing kernels via the mature plant^1^,^3^,^4^,^5^. The diseases caused by *F. verticillioides* include seedling blight, stalk rot, root rot, kernel rot, ear rot, and seed rot^5^,^6^. *F. verticillioides* infection can result in decreased grain yields, poor grain quality, and contamination by the mycotoxin fumonisin^7^. The fumonisins produced by *F. verticillioides* are known to cause a variety of diseases when ingested by animals, and have been implicated in human carcinogenesis and neural tube defects, as well as in plant diseases^6^.

Development of maize varieties that possess seeds with disease resistance is an effective way to improve resistance to soil-borne diseases. Increased FSR resistance could reduce the incidence of the above-mentioned diseases caused by *F. verticillioides*. At present, the majority of studies concerning *F. verticillioides* resistance have focused on *Fusarium* ear rot (FER) in maize, and there is presently no report on the genetic architecture of FSR resistance. Previous studies have identified a number of quantitative trait loci (QTL) associated with FER resistance present on the majority of the ten chromosomes in maize^8^,^9^,^10^,^11^,^12^,^13^. However, the effects of these QTLs were relatively small and not consistent between populations due to the genetic heterogeneity, the inability to detect the minor effects of all variants, and the influence of environmental factors.

Genome-wide association study (GWAS) is a useful tool for identifying candidate loci associated with numerous traits in many plants^14^. GWAS is especially useful when combined with QTL mapping in order to map and validate loci for complicated traits because the combination of these two methods mitigates the limitations of either method performed alone^15^. This strategy has been successfully used to identify QTLs and candidate genes for various ecological and agricultural traits in soybeans^16^, rice^17^, wheat^18^, rapeseed^19^, sunflowers^20^, and other plants. With the development of the large-scale molecular marker detection technology, a diverse collection of samples of maize inbred lines, landraces, and teosintes have been genotyped through genotyping by sequencing (GBS) performed by Cornell and CIMMYT^21^,^22^,^23^. The combination of these data in GWAS and linkage analysis has proved to be useful in detecting and identifying the main resistance loci in maize. However, this combined approach has seldom been used to study *F. verticillioides* resistance in maize.

In the present study, we combined GWAS and linkage mapping to identify the candidate genes and QTLs associated with FSR resistance in maize. The three objectives of this study were to (1) identify the gene(s) and QTL(s) that significantly affect *F. verticillioides* resistance in seeds, (2) validate these resistance-related QTL(s) and gene(s), and (3) compare the expression levels and structural differences of the candidate genes between the two parental lines.

## Results

### Phenotypic characteristics of FSR resistance in association mapping panels and the bi-parental RIL population

In the bi-parental RIL population, the parental line BT-1 was highly resistant to *F. verticillioides,* with a rating scale of 1.67, while the parental line N6 was highly susceptible, with a rating scale of 6.00. A wide range of *F. verticillioides* resistance was found in the progenies of the RIL population; the rating scale ranged from 0.28 to 6.07, with a mean of 3.56 in the combined analysis (Table 1). The frequency distribution of the disease index in the 174 RIL lines showed a skewed normal distribution (Supplementary Fig. S1), whilst it showed a normal distribution in association mapping panels (Supplementary Fig. S2). The rating scale for FSR resistance of 174 RIL lines ranged from 0.50 to 6.52 (Table 1). Those lines in disease grades 0–1, 1–2, 2–3, 3–4, 4–5, 5–6, and 6–7 accounted for 4.15%, 4.61%, 18.89%, 36.87%, 26.73%, 6.45%, and 2.30% of the total association mapping panels, respectively. The lines CML424 and CML156 from the association mapping panels had the lowest and the highest FSR resistance rating scale of 0.48 and 6.55, respectively. In the combined analysis, the heritability was found to be 0.79 and 0.93 in the association mapping panel and RIL population, respectively.

### QTL mapping for FSR resistance

Eight QTLs were identified as related to FSR resistance, which were distributed on maize chromosomes 1, 2, 3, 4, 5, and 8, respectively (Supplementary Fig. S3). The percentage of phenotypic variation explained by these QTLs ranged from 4.07% to 12.06% (Table 2), and *qFR2–1*, which explained 12.06% of the total phenotypic variation, was found to have the largest effect. Among these QTLs, the favourable alleles of *qFR1, qFR2–1,* and *qFR4-1* are derived from the resistant parent BT-1, whilst the remainder are derived from the susceptible parent N6.

### Population structure analysis, LD decay, and association mapping analysis

The GBS version 2.7 build generated 955,690 single nucleotide polymorphisms (SNPs) for each genotyped sample, of which 955,120 were assigned to chromosomes 1–10; the remaining 570 SNPs could not be mapped to any of the ten maize chromosomes. For these 955,120 SNPs, 224,152 of them were kept for future analysis of their missing rate, which was less than 10%, and of their minor allele frequency, which was greater than 0.05. Population structure analysis of a random selection of 5000 SNPs on the association mapping panel showed that the estimated log probability of the data (LnP(D)) sharply increased between K = 1 and K = 3, and then slowly increased after K = 4 (Supplementary Fig. S4). Hence, K = 3 was assumed to be the most appropriate value to use for grouping as the peak value of Delta k (ΔK) appeared when K = 3(Supplementary Fig. S4). As shown in Supplementary Table S1, the majority of the lines (128) were assigned to group 1, which included lines from CIMMYT (127) and the P group (1). The second group consisted of 52 lines, which mainly came from Chinese inbred lines of Tangsipingtou group (TSPT) (23), bi-parental RILs (28), and BT-1. Group 3 consisted of 34 lines from Reid group (16), P group (12), Lancaster group (5), and Luda Red Cob group (1). The remaining three genotypes were classified into a mixed group, as the probability of them having membership in one of the three groups was <60%. The results of principal component analysis by 224,152 SNPs according to the PC1 (x-axis) relationship analysis also indicated that the association panel were mainly divided into 3 groups (Supplementary Fig. S5).

**Figure 1:**
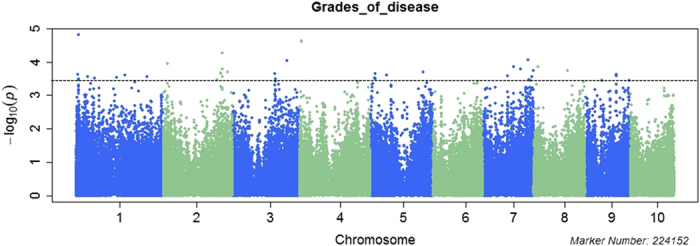
Manhattan plots of GWAS for the grades of disease resistance to *F. verticillioides* seed rot in maize. Plots above the imaginary line show the genome-wide significance with a moderately stringent threshold of - log (100/224,152). The horizontal axis shows the physical positions on the ten chromosomes. The vertical axis shows the value of - log (P).

**Figure 2:**
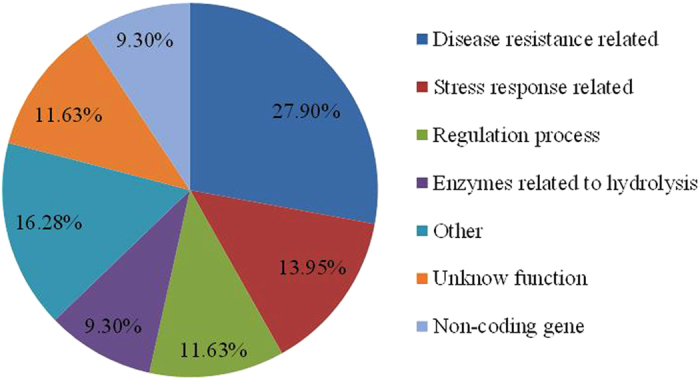
Functional category annotations for 43 candidate genes and their respective percentages, identified via GWAS as significantly associated with resistance to *F. verticillioides* seed rot in maize.

Linkage disequilibrium (LD) for the association mapping panels was measured using 224,152 SNPs (Supplementary Fig. S6). LD decay varied across the ten chromosomes, as well as across different genetic regions within the chromosomes. The LD decay distance across all ten chromosomes ranged from 10 kb to 100 kb when the cut off value of r^2^ was set as 0.1. The MLM+K+PCA and MLM+K+Q were used to perform the association analysis. Association results with the two models were shown as quantile-quantile plots of estimated –log 10(p) (Supplementary Fig. S7).The QQ Plot of the two models showed that the observed P value (the statistical significance of detection for each SNP) was lower than the expected P value at a threshold greater than three. As a result, some loci with lower significance may not have been detected, but this should not have affected the identification of loci significantly associated with FSR resistance. In addition, the observed P value of MLM+K+PCA model was more closed to the expected P value than that of the MLM+K+Q model under the threshold. Considering all the filtered SNP (224,152) were used in the PCA analysis, so we use the result of MLM+K+PCA model for further analysis As shown in Fig. 1, in the Manhattan plots of MLM+K+PCA model for the grades of disease of seed resistance to *F. verticillioides*, 57 SNPs were identified as significantly associated with FSR resistance when using P < 4.46 × 10^−4^ as the threshold for declaring significance in a MLM whole genome wide scanning. These 57 SNPs were distributed on all of the maize chromosomes except for chromosome 10, and the percentage of phenotypic variation explained ranged from 6% to 10%. After examining the physical position of these SNPs, we found that 37 were located in the coding regions, 19 were close to the candidate genes, and one was not related to any genes (Table 3 and Supplementary Table S3). Finally, 43 candidate genes were identified during association mapping analysis of FSR resistance and most of them had functions related to disease resistance, stress response, regulation processes, or hydrolysis enzymes; 27.9% of these genes were involved in different plant disease response pathways (Fig. 2).

### Candidate genes identified by combining QTL mapping and GWAS

Through comparison with the result of GWAS, the associated SNPs of chr1.S_6433914, chr3.S_187896997, chr5.S_56372680, and chr8.S_172132198 were co-localized with the intervals of QTL *qFR1, qFR3, qFR5,* and *qFR8*, respectively. The genes corresponding to chr1.S_6433914, chr3.S_187896997, and chr5.S_56372680 were *GRMZM2G0081223, AC213654.3_FG004,* and *GRMZM2G099255*, respectively, whilst SNP chr8.S_172132198 was located in the non-coding region. Therefore, *GRMZM2G0081223, AC213654.3_FG004,* and *GRMZM2G099255* were detected in both QTL mapping and GWAS, suggesting that they are candidate genes for FSR resistance.

**Figure 3:**
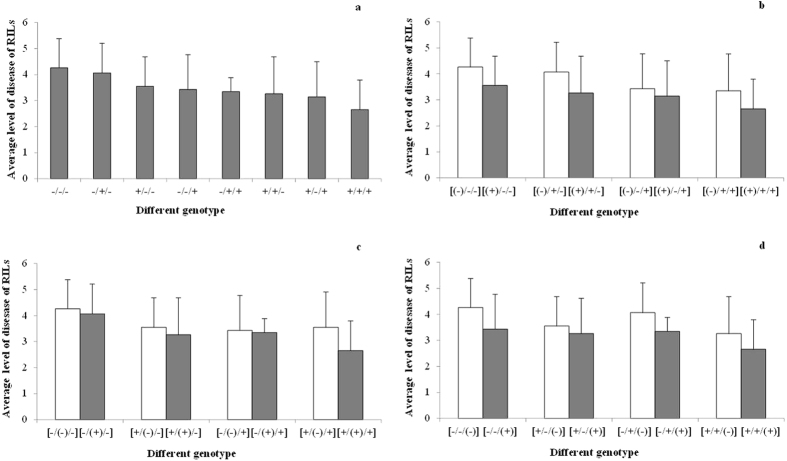
Influence of different genotype combinations of three loci on the resistance of the RIL materials. [−or +/−or +/−or +] represent whether the 3 genes of RILs on chr.1/chr.3/chr.5 are resistant (+) or susceptible (−). The resistant (+) gene and the susceptible (−) gene on chr.1 come from the BT-1 and N6 respectively; the resistant (+) gene and the susceptible (−) gene on chr.3 and 5 come from the N6 and BT-1, respectively. The horizontal axis represents the different genotypes of the 3 candidate genes on chromosomes 1, 3, and 5 as resistant (+) or susceptible (−). The vertical axis shows the average level of disease of RIL materials with the same genotype as the 3 candidate genes on chromosomes 1, 3, and 5.

The effects of the three genes *GRMZM2G0081223, AC213654.3_FG004,* and *GRMZM2G099255* were validated in the RIL population using their co-localized or closely flanked SSR markers, which were flanked by umc1291 to umc1106, umc1744 to bnlg197, and umc1935, respectively. The results showed that chr5.S_56372680 had the most obvious effects in relation to resistance, followed by chr1.S_6433914; chr3. S_187896997, however, had almost no effect. The co-existence of the three resistant loci showed the highest effect on the resistance in the RIL (Fig. 3a), and the resistant levels were as follows: [+/+/+] > [+/−/+] > [+/+/−] > [−/+/+] > [−/−/+] > [+/−/−] > [−/+/−] > [−/−/−]. The resistant loci of chr1.S_6433914 or chr5.S_56372680 could obviously enhance the resistance effects of any combined loci (Fig. 3b and d). In the absence of the chr1.S_6433914 and chr5.S_56372680 loci or the single locus chr5.S_56372680, the resistance locus chr3.S_187896997 had little effect on the phenotypic variation. On the other hand, the resistance locus of chr3.S_187896997, when co-existing only with locus chr1.S_6433914 or both the chr1.S_6433914 and chr5.S_56372680 loci, could affect resistance. These results indicate that *GRMZM2G0081223* and *GRMZM2G099255* can function independently in resistance to *F.verticillioides*, whilst *AC213654.3_FG004* may be downstream of *GRMZM2G0081223*.

Furthermore, the locus containing the *GRMZM2G0081223* gene was confirmed using near-isogenic line (NIL) populations. We developed the NILs by introgressing the target locus chr1.S_6433914 from BT-1 using markers umc1292 to umc1106. The NIL containing locus chr1.S_6433914 showed significantly enhanced seed resistance to *F. verticillioides* compared to other NILs with only the N6 background (Table 4), indicating that the locus chr1.S_6433914 contributed significantly to FSR resistance in the NIL background.

### Comparison of candidate genes between the parental lines of BT-1 and N6

To compare the genomic differences of these candidate genes between the two parental lines, BT-1 and N6, we re-sequenced them and obtained a total of 137.78 Gbp of raw data. After filtering out low quality and short reads, 130.90 Gbp of clean reads were kept, with an average genomic coverage of 31× for each parental line. Of the clean reads obtained, 90.98% were mapped on the reference genome, covering 89.36% of the total reference genome. A total of 7,292,643 SNPs and 1,546,933 indels were identified between BT-1 and N6.

Using these sequence variations, we compared the differences in the genomic regions of the three candidate genes. The genomic size of *GRMZM2G008122* was 5986 bp, containing 12 exons and 11 introns. Compared to BT-1, the *GRMZM2G008122* gene (accession number: KT947107) in N6 had 53 single base substitutions, 9 insertions, and 6 deletions, causing amino acid changes in the 144^th^, 260^th^, 488^th^, 489^th^, and 928^th^, and insertion in the 490^th^–494^th^ (Supplementary Fig. S9). The protein encoded by *GRMZM2G008122* was a plasma membrane ATPase-like protein with two structural domains inside: an E1-E2_ATPase domain and a hydrolase domain. The gene of *AC213654.3_FG004* was a 1089 bp long polypeptide in BT-1 and N6 (accession number: KU645845 and KU645846, respectively), and the structural prediction of *AC213654.3_FG004* revealed that it contains an F-box like domain. Compared to BT-1, there were seven single base substitutions in the N6 genome, which resulted in two replacements in the 67^th^ and 267^th^ amino acids and an insertion from the third to tenth amino acid (Supplementary Fig. S10). The candidate gene of *GRMZM2G099255* had two transcripts (GRMZM2G099255_T01 and GRMZM2G099255_T02). The ORF of GRMZM2G099255_T01 contained 10 exons and 9 introns, which showed no differences between the two parental lines, whilst the ORF of GRMZM2G099255_T02 contained 7 exons and 6 introns, which had 64 replacements, 5 insertions, and 2 deletions in N6 (accession number: KT947108). As a result, the amino acid of N6 had 15 replacements at the positions of the 240^th^, 259^th^, 285–294^th^, and 296–298^th^, and 2 insertions at the positions of the 299^th^ and 300^th^ (Supplementary Fig. S11). GRMZM2G099255_T02 encoded a beta-1, 6-N-acetylglucosaminyl transferase enzyme. In line BT-1, a trans-membrane helix region spanned the 13–32^th^ amino acids, whereas there was a signal peptide in N6 of the 1–28^th^ amino acids.

### Expression analysis of candidate genes

**Figure 4:**
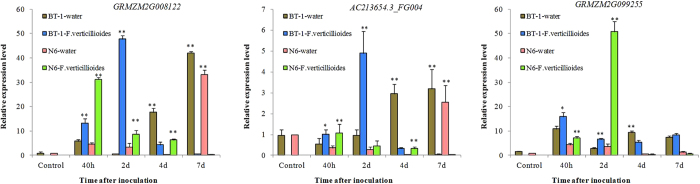
Quantitative real-time PCR of three candidate genes at different times for BT-1 and N6 after being inoculated with water and *F. verticillioides*. * and ** denote significant differences in the comparison between water treatment and *F. verticillioides* treatment at p = 0.05 and p = 0.01, respectively (LSD-ANOVA significance test).

To further confirm the candidate genes, we analysed the expression levels of the three genes using qRT-PCR in seeds of the two parental lines after 40 h, 2 days, 4 days, and 7 days of inoculation with *F. verticillioides.* The results showed that all three genes showed significantly up-regulated expression compared to the water treatment. The highest expression levels of *GRMZM2G0081223* and *AC213654.3_FG004* were obtained at 2 days after inoculation with *F. verticillioides* in BT-1 seeds, and at 40 h after the treatment in N6 seeds. Both the genes had their highest expression levels at 7 days after inoculation with water in both BT-1 and N6 (Fig. 4), indicating that the expression of these two genes is correlated with the early period of pathogen infection. *GRMZM2G099255* had its highest expression level at 2 days after inoculation with *F. verticillioides* in N6 seeds, and at 40 h after treatment in BT-1 seeds (Fig. 4). *GRMZM2G0081223* and *AC213654.3_FG004* had the same expression trend, which differed from that of *GRMZM2G099255*, indicating *AC213654.3_FG004* and *GRMZM2G0081223* may be in the same genetic networks.

## Discussion

Accurate phenotype identification is the basis for research on genetic resistance to plant diseases. To date, there are few reports on FSR except for a number of studies on the induction and control of maize seed rot^24^,^25^. To our knowledge, there remain no reports on the identification of FSR resistance and its genetic mapping in maize. Taking evaluation methods for resistance to *Aspergillus flavus* in peanuts as a reference^26^, we inoculated maize seed with *F. verticillioides* in a lab under unified spore concentration standards and inoculation conditions to reduce environmental error. Tissue observation revealed that the pathogenic fungus infected maize seed embryos at the tips and crowns of the seeds, and that the degree to which the embryo inside the seed was infected was closely related to the visual symptoms on the seed coat. Before phenotype identification of the two genetic populations, we compared the grade of disease between parent lines BT-1 and N6 treated with different spore concentrations (1 × 10^3^, 1 × 10^4^, 1 × 10^5^, 1 × 10^6^, and 1 × 10^7^) and over different inoculation periods (1d, 2d, 3d, 4d, 5d, 6d, 7d, and 8d). Results showed that the BT-1 line had better disease resistance than N6 in all experimental conditions (Supplementary Fig. S13). Using the same conditions (temperature 28 °C, humidity 70%, and maintained in darkness) for all inoculated populations, we chose a spore concentration of 1 × 10^5^ and an inoculation period of seven days as the initial inoculation concentration and survey period, respectively, as these conditions were conducive to the greatest difference observed between the parental lines. Seven days inoculation was deemed appropriate to analyse infection because the spread of hyphae on the seed coat largely stalls during this period. The FSR resistance rating scale was found to have a normal distribution in the association mapping panel, while the rating scale in RILs showed a skewed normal distribution, indicating that phenotyping methods based on direct inoculation reflected the comprehensive resistance of RILs.

**Figure 5:**
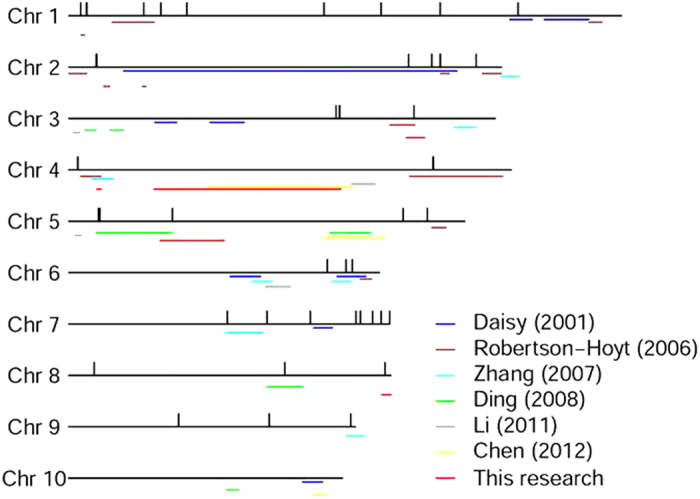
Comparison of QTLs for *Furiasum* ear rot resistance detected by previous reports and *F. verticillioides* seed rot resistance in this study. Vertical lines represent the 57 significant associations with seed resistance to *F. verticillioides* this study found in GWAS; horizontal lines of different colours represent different QTL intervals of *Fusarium* ear rot identified by previous reports; red horizontal lines represent the QTL interval of *F. verticillioides* seed rot resistance identified in this study.

In a previous study, Munkvold *et al*.^4^ confirmed that systemic *F. verticillioides* infection derived from maize seeds can contribute to kernel infection^5^. Therefore, in addition to affecting maize yield and seed quality, *F. verticillioides* infection can also be detrimental to grain quality. To date, marker-trait associations have had only small additive effects, and these effects have been in consistent between studies, as FER is easily influenced by environmental conditions^7^. Each of the loci associated with improved ear rot resistance identified by Zila *et al*.^7^,^27^ had small allelic effects. Our results are similar, as we found phenotypic contributions ranging from 6.0% to 10.4%. Using linkage mapping, the highest contribution was found to be 12.06%. Few high contributions to FER have been found in previous reports. In the process of detecting QTLs related to FSR resistance, we have been performed the permutation tests, and the threshold of LOD value was 3.02 under type 1 error as 0.05. Previous studies of *F. verticillioides* resistance in maize showed that the score of LOD was set 2–3^10^,^11^,^12^,^13^. In order to compare our results with those reported elsewhere, we set the LOD value at 2.5. Concerning P values of GWAS, the threshold is usually set at 0.05/number of SNPs in the correlation analysis in order to control the error rate, according to the Bonferroni correction method. The Bonferroni test is applied based on the assumption of independence between markers^28^, but this threshold is typically very strict as GWAS is hypothesis-generating^29^,^30^. As for the high-density SNPs and linkage disequilibrium between markers, the statistical threshold for GWAS was decreased to obtain the true associations for plants^30^. Considering that the Bonferroni correction is too strict for correcting the high-density marker, especially for those quantitative traits controlled by many small-effect loci, we applied the 100/number of SNPs to correct the denominator of the Bonferroni correction, based on the results of the LD analysis. This process enables us to find more significant sites for further comparison with QTLs in linkage mapping. After comparing the 57 associated SNPs and 8 QTLs of FSR resistance with the previously reported QTLs of FER in maize, it was found that 20 of the associated SNPs were localized on the same intervals as previous reported QTLs, and that 5 of the QTLs identified in this study were co-localized with the QTLs identified in previous reports (Fig. 5)^8^,^9^,^10^,^11^. The above evidence suggests that FER and FSR resistance may share similar mechanisms to that of resistance to *F. verticillioides*. However, we also evaluated the FER resistance of 100 randomly selected inbred maize lines from the association panel by artificially inoculating these lines using the nail-punch method. Based on the correlation analysis of FER and FSR resistance for 100 maize lines, we found that there was no significant correlation between the two kinds of disease as shown in Supplementary Fig. S14 (R^2^ = 0.0218). Although *F. verticillioides* infection in seeds can cause FER to some extent^5^, the pathogenic mechanisms and resistance pathways differ between the two diseases.

Maize germplasm in China is mainly divided into a number of heterosis groups including TSPT group, Reid group, P Group, Lancaster group, Luda Red Cob group, and some tropical and subtropical groups. To dissect the genetic architecture of FSR resistance in maize, 217 inbred maize lines were selected for association analysis regarding 224,152 SNPs. These SNPs were unevenly distributed over the ten maize chromosomes and displayed high-density of one SNP per 9.22 kb, which was beneficial in identifying the SNPs associated with resistance to *F. verticillioides*. Structural analysis of the genotypes of 217 inbred maize lines showed that these association mapping lines were mainly divided into three groups. Phylogenetic tree of the association panel showed that the CYMMYT lines were divided into several distinct subgroups (supplementary Fig. S11). They were generated from different races with wide genetic variability through tracing the consanguinity. The detail pedigree information can be found on the CIMMYT Dataverse Network. In the Huang-Huai-Hai region of China, TSPT lines and lines not related to TSPT (e.g. Reid and the P group) are often used in heterosis patterns, which lead to genetic mixing. As a result, some different lines may be categorized into the same groups, such as BT.N6pop and TSPT were grouped into the same group. PCA analysis showed that positions of the BT.N6 population and the TSPT group were identical and grouped into the same group according to the PC1 (x-axis) relationship analysis. Using the PC2 factor (y-axis) analysis, the BT.N6 population and TSPT were found to be divided into two small subsets of the same group. Considering that PC1 is the main impact factor for population structure analysis, we grouped the optimum population into three groups, and BT.N6pop and TSPT group were grouped into the same group.

As maize has high genetic diversity and rapid LD decay, GWAS has been successfully used to analyse the genetic architecture of many complex traits, such as resistance to head smut^31^, oil biosynthesis^32^, seedling root development^33^, and resistance to northern corn leaf blight^34^. In the present study, 57 SNPs related to 43 genes were detected in association with resistance to FSR. Twelve of these SNPs were predicted to also have some function in fungal infection resistance, such as the WRKY DNA-binding protein and serine/threonine protein kinesis^35^. In a previous study, Zila *et al*.^7^ conducted a genome-wide association study for FER resistance in a panel of 1,687 diverse inbred lines using 200,978 SNPs, and identified seven SNPs in six genes associated with FER resistance; none of the genes they identified had a predicted function in relation to disease resistance^7^. The SNPs associated with FSR resistance identified in this study were different to those reported by Zila *et al*.^7^,^27^, except in the case of the loci on chromosome one, which were similar to those reported by Zila *et al*.^7^,^27^. According to the function classification of the candidate genes in this study, several genes encode proteins which are known to be involved in plant disease responses. For example, the candidate gene *AC213654.3_FG004* contains an F-box like structural domain. F-box genes are one of the most abundant gene super families in plants, and their protein products are involved in ubiquitination and degradation^36^. Zila *et al*.^7^ also identified two of the four SNPs significantly associated with FER resistance through a GWAS localized to exon of an F-box domain gene, suggesting that F-box genes play an important role in *F. verticillioides* disease resistance in maize. The structural domain of GRMZM2G099255_T02 is a branch of beta-1, 6-N-acetylglucosaminyl transferase enzymes that belonged to the glycosyl transferase family. Studies on *Arabidopsis thaliana* have confirmed that glycosyl transferase can not only modify plant hormones to regulate plant disease resistance, but is also a necessary factor in plant allergic reactions against disease^37^,^38^. Lorenc-Kukula*et al*.^39^ reported that over-expressing the glycosyl transferase (UGT) protein SsGT1 derived from *Solanum sogarandinum* in flax resulted in increased resistance to *Fusarium* infection. Functional verification through genetic transformation of the candidate gene will be required to clarify the functions of the pathway to FSR resistance in maize in the future.

## Material and Methods

### Plant Material

The inbred parent BT-1, which originated from the Suwan germplasm of Thailand and elite inbred line 8085 of China, is one of the parental lines of the elite hybrid Guoshenyu2005026. The inbred parent N6 is an improved version of another inbred line huangzao4, which was developed from TSPT, thus it has weak disease resistance but high combining ability; it is an overall good plant type, with a short growth period and high stress resistance. The two parental inbred lines, BT-1 and N6, have been identified as lines that show the highest resistance and highest susceptibility to *Fusarium* ear rot (FER), respectively. Therefore, a RIL population containing 174 F_9:10_ lines were developed from these two lines for QTL mapping analysis using the single seed descent method for QTL. The NIL used in this study was also developed by crossing the recipient line N6 with the donor line BT-1, through four cycles of advanced backcrosses. A novel panel consisting of 217 diverse maize inbred lines was developed for evaluating FSR resistance used for GWAS in this study. Based on this pedigree information, the completed panel mainly came from the lines of Reid group, Lancaster group, Tangsipingtou (TSPT)group, and tropical heterotic “A” and “B” groups from International Maize and Wheat Improvement Centre, as well as a RIL derived from the bi-parental lines of BT-1 and N6. All the plant materials are reserved at the Agronomy College of Henan Agricultural University in China.

### Inoculation and phenotype analysis

For each material tested through QTL mapping and GWAS, a uniform number of seeds were selected and sterilized with 75% ethyl alcohol for 30 s and 3% sodium hypochlorite for 15 min. Then, the seeds were washed three times with sterile water. *F. verticillioides* used in the study was isolated from naturally infected kernels using the single-spore isolation method^40^. The treated seeds were inoculated with a 1 × 10^5^ *F. verticillioides* spore suspension for 12 h. The inoculated seeds were then washed with sterile water three times and their surfaces were dried with filtered paper. For each tested sample, 10 seeds were randomly selected for phenotypic evaluation after co-inoculation with 1 × 10^5^ *F. verticillioides* spore suspension, and so were put on a Petri dish with 4 ml water containing 2 pieces of filter paper; 2–3 replications were conducted. The inoculated Petri dishes were stored in the illumination incubator (BoXun, China) at 28 °C in the darkness. One week following inoculation, visual evaluations of disease severity were assessed by screening the number of infected seeds and the percentage of the kernels displaying signs of disease in each Petri dish. A rating scale from 0 to 7 was used to assess the severity of seed infection; detailed information about the rating scale can be found described in Supplementary Table S4. Examples of samples rated from 0 to 7 are shown in Supplementary Fig. S12.

The original seeds of the RIL population and the GWAS panel were collected from Hainan Province in China. Seeds of each material were inoculated with *F. verticillioides* three times for the RIL population, and twice for the GWAS panel. During combined analysis, each time was treated as an environment, and the best linear unbiased predictors (BLUE) for each line were calculated using PROC MIXED procedure of SAS software. Broad sense heritability (H^2^) was calculated in SAS using the equation: H^2^ = 
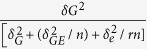
, where is the genotypic variance, 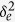 is the genotype environment variance, 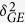 is the residual error variance, and n and r are the number of environments and replications, respectively^34^. Normality test of association mapping panel and the RIL population was performed using the Shapiro-Wilk test in SAS.

### QTL mapping

In our previous study^12^, a genetic map was constructed using 250 RILs and 207 SSR markers which spanned a total length of 1,820.8 cM, with an average distance of 11.7 cM between markers. This map was used to detect the QTLs in resistance to *F. verticillioides* in our current study. Sequences of oligonucleotide primers for SSR markers were obtained from the Maize Genetics and Genomics Database, available at its website (http://www. maizegdb.org). We used the 174 RILs and the published genetic linkage map to locate the QTLs related to the disease resistance in maize. Linkage mapping analysis were conducted in QTL IciMapping version 4.0^41^, with the BLUE value of each tested line used as phenotypic data for multi-location QTL analysis across the seed original sources and replications. The scanning step was 1 cM, and only one-dimensional QTL scanning was conducted, which means only additive (a) and dominance (d) effects for each QTL were estimated. The LOD threshold used for declaring putative QTL was defined as 2.5.

### Genotyping by sequencing and association mapping

All the DNA samples tested in GWAS were sent to Cornell University Biotechnology Resource Centre for genotyping, where the GBS protocol described by Elshire *et al*.^22^ was performed. The *ApeKI* restriction enzyme was used for DNA digestion, and 96 samples were multiplexed for library construction and sequencing. SNP calling was performed using TASSEL 5.0 GBS Discovery Pipeline, with B73 as the reference genome, and imputation was conducted using the FILLIN method in TASSEL 5.0; the imputed dataset was used for further analysis.

Of the SNPs generated by the GBS version 2.7, 5,000 were randomly selected for population structure analysis. The analysis was performed in software Structure (version 2.3.4) using the Markov Chain Monte Carlo model^42^,^43^,^44^, where the burn-in period and Markov Chain Monte Carlo were set to 10,000 and,100,000, respectively, using an admixture model-based clustering method. The number of K clusters ranged from 1 to 12, with the model run four times for K ranging from 1 to 8, and two times for K ranging from 9 to 12. The number of clusters (true K value) was determined according to the *ad hoc* statistic delta K (*ΔK*)^45^. Each inbred line was assigned to a particular cluster when the genetic similarity ratio within each cluster exceeded 60%. If an inbred line had a similarity ratio of less than 60% to all groups, it was sorted into the mixed cluster. Principal component analysis (PCA) was conducted in TASSEL software V5.0^46^ by using all filtered SNPs which was further used as covariate for association mapping analysis to reflect the population structure. The phylogenetic tree of association panel based on neighbour joining (NJ) method was generated in TASSEL software V5.0.

Average LD between SNPs on each chromosome was measured in TASSEL software V5.0. The squared Pearson correlation coefficient (r^2^) between vectors of SNP alleles was used to assess the level of LD decay on each chromosome. Association mapping analysis was conducted in TASSEL using the mixed linear model MLM+Kinship+PCA and MLM+Kinship+Q. The threshold for declaring significant associations was 100/number of SNPs. Manhattan plots and quantile-quantile plots were drawn using the R package.

### DNA sequencing and sequence comparison

Genome resequencing of BT-1 and N6 was carried out following the standard Illumina protocol, and the library was used for paired-end sequencing on the Illumina HiSeq2500 analyser. After removing short reads and low quality reads, the clean reads were used for mapping to maize reference genome B70 (Zea_AGPv3_B70, http://www.maizegdb.org/assembly/) using software BWA^47^. SNPs and small indels detected from the alignments were called using Samtools^48^, and output was given in pileup format. The quality score of the SNPs was assigned by Samtools to evaluate the reliability of SNP calling based on the Phred-scaled probability that the consensus is identical to the reference. The SNPs and small indels between two parental lines were detected using the GATK software tool package^49^, and the reliable SNPs and small indels were noted and predicted using SnpEff software^50^. By comparing the resequencing of the two parental lines and reference genome, Insertion (INS), Deletion (DEL), Inversion (INV), Intra-chromosomal Translocation (ITX), and Inter-chromosomal Translocation (CTX) were detected using BreakDancer software^49^.

### RNA isolation and qRT-PCR

Seeds of BT-1 and N6 were inoculated with *F. verticillioides* and watered for40 h, 2 days, 4 days, and 7 days, and then used to analyse the expression profiles of candidate genes. The total RNA of the seeds was isolated using Plant pillar RNA out 2.0 (TIANDZ, China); the first strand of cDNA was synthesized using the 5× All-In-One RT Master Mix (abm, Canada). The beta-actin gene was selected as the endogenous gene. Gene expression was determined through qRT-PCR assays, using the system of EvaGreen qPCR Mastermix (abm, Canada) with designed primes (Supplementary Table S5) on the Real-time quantitative PCR chromo4 (Bio-Rad, America). The expression level ratio was calculated for each sample as 2^[−△CT (test)−△CT(calibrator)]^, where ΔCT (test) = CT (target, test) − CT (Actin, test), and ΔCT (calibrator) = CT (target, calibrator) − CT (Actin, calibrator). The expression of N6 seeds that had not been inoculated was used as a calibrator.

### Ethical standards:

The authors declare that the experiments comply with the current laws of the countries in which the experiments were performed.

## Additional Information

**How to cite this article:** Ju, M. *et al*. Dissecting the genetic architecture of *Fusarium verticillioides* seed rot resistance in maize by combining QTL mapping and genome-wide association analysis. *Sci. Rep.* **7**, 46446; doi: 10.1038/srep46446 (2017).

**Publisher's note:** Springer Nature remains neutral with regard to jurisdictional claims in published maps and institutional affiliations.

## Supplementary Material

Supplementary Tables and Figures

## Figures and Tables

**Table 1:** Mean and range of grades of disease for the association mapping panel and the RIL population together with variance components (σ_g_^2^, σ_ge_^2^, σ_e_^2^), repeatability, and heritability (H^2a^) estimates for *F. verticillioides* resistance in different environments.

Population	Environments	Mean ± SD	Range	σ_g_^2^	σ_ge_^2^	σ_e_^2^	H^2a^
RIL Population	I	3.28 ± 1.74	0.00–7.00	7.79		0.17	0.99
	II	3.55 ± 1.42	0.00–6.00	3.40		0.18	0.98
	III	3.88 ± 1.68	0.00–7.00	5.61		0.24	0.98
	Combined	3.56 ± 1.23	0.28–6.07	11.66	2.54	1.04	0.93
Association mapping lines	I	3.14 ± 1.76	0.00–7.00	8.84		0.22	0.99
	II	4.09 ± 1.59	0.00–7.00	5.11		0.26	0.96
	combined	3.54 ± 1.18	0.50–6.52	8.89	4.41	0.24	0.79

**Table 2:** QTL mapping for *F. verticillioides* seed rot resistance.

Chromosome	Position	Marker interval	LOD	PVE (%)	Additive effect	QTL
1	7	umc1106-bnlg1014	5.95	10.14	−0.39	*qFR1*
2	77	umc1776-bnlg1064	6.96	12.06	−0.43	*qFR2-1*
2	89	bnlg1018-phi083	4.05	6.7	0.32	*qFR2-2*
3	121	umc1644-bnlg197	2.57	4.07	0.25	*qFR3*
4	53	umc2280-umc2281	3.27	5.34	−0.3	*qFR4-1*
4	85	umc1964-mmc0371	3.04	5.09	0.28	*qFR4-2*
5	72	umc1935-umc2298	3.68	5.9	0.31	*qFR5*
8	113	umc1384-phi080	2.84	4.79	0.27	*qFR8*

**Table 3:** Physical positions of partial SNPs significantly associated with *F. verticillioides* seed rot resistance and the predicted function or homology of adjacent candidate genes.

Marker	P-value	Resistant allele	R^2^	Gene	Annotation	Class
Chr.1_6433914	2.36E-04	C	0.074	GRMZM2G008122	(AHA3, ATAHA3, HA3) H( + )-ATPase 3	Stress response related
Chr.1_9580795	3.28E-04	T	0.066	GRMZM2G415390	Disease resistance-responsive family protein	Disease resistance related
Chr.1_9807793	1.54E-05	C	0.095	GRMZM2G009818	Leucine-rich repeat transmembrane protein kinase family protein	Disease resistance related
Chr.1_9811052	3.52E-04	C	0.084			
Chr.1_40812382	2.83E-04	T	0.076	GRMZM5G891990	(ATPUB13, PUB13) plant U-box 13	Disease resistance related
Chr.1_50100218	4.43E-04	G	0.059	GRMZM2G394212	AD/NAD(P)-binding oxidoreductase family protein	Disease resistance related
Chr.1_64239355	3.10E-04	A	0.073	GRMZM2G147698	(ATMYB61, MYB61) myb domain protein	Stress response related
Chr.1_138914021	2.95E-04	G	0.063	GRMZM2G099010	alpha/beta-Hydrolases superfamily protein	Enzymes related to hydrolysis
Chr.1_169988783	2.55E-04	G	0.088	GRMZM2G155314	ankyrin repeat family protein	Stress response related
Chr.1_244559899	2.86E-04	G	0.066	GRMZM2G120085	Subtilase family protein	other
Chr.2_14836550	1.13E-04	G	0.073	GRMZM2G303118	P-loop containing nucleoside triphosphate hydrolases superfamily	Enzymes related to hydrolysis
Chr.2_184967793	3.49E-04	A	0.069	GRMZM2G414252	(HEC1) basic helix-loop-helix (bHLH) DNA-binding superfamily protein	Regulation process
Chr.2_197597371	2.22E-04	G	0.071	GRMZM2G117865	S-locus lectin protein kinase family protein	Disease resistance related
Chr.2_197598070	2.16E-04	G	0.068			
Chr.2_202176728	2.86E-04	T	0.075	GRMZM2G154864	Transducin/WD40 repeat-like superfamily protein	
Chr.2_202178253	5.37E-05	A	0.08			Disease resistance related
Chr.2_202178298	1.62E-04	G	0.071			
Chr.2_221753785	2.04E-04	A	0.076	GRMZM2G106560	(ATWRKY75, WRKY75) WRKY DNA-binding protein 75	Disease resistance related
Chr.3_145476172	2.27E-04	T	0.068	GRMZM2G397948	ubiquitin-protein ligases	Disease resistance related
Chr.3_147411182	3.81E-04	T	0.066	GRMZM2G138342	(ATNADK-1, NADK1) NAD kinase 1	
Chr.3_147411924	3.22E-04	A	0.067			Disease resistance related
Chr.3_187896997	9.22E-05	G	0.088	AC213654.3_FG004	(EDL3) EID1-like 3	Stress response related
Chr.4_4867002	2.51E-05	T	0.098	GRMZM2G468260	RING/U-box superfamily protein	Regulation process
Chr.4_4867006	2.35E-05	T	0.099			
Chr.4_198337279	3.87E-04	T	0.06	GRMZM2G071405	Pentatricopeptide repeat (PPR-like) superfamily protein	other
Chr.4_198337821	4.31E-04	T	0.062			
Chr.4_198560823	3.91E-04	C	0.098	AC234156.1_FG005	(FLA7) FASCICLIN-like arabinoogalactan 7	other
Chr.5_16096144	3.05E-04	G	0.066	GRMZM2G160619	Leucine-rich repeat protein kinase family protein	Disease resistance related
Chr.5_16844527	2.32E-04	T	0.082	GRMZM2G077828	(ATCNGC7,) cyclic nucleotide gated channel 7	
Chr.5_16844540	2.32E-04	T	0.082			Disease resistance related
Chr.5_16845040	3.11E-04	A	0.082			
Chr.5_16845047	3.19E-04	A	0.082			
Chr.5_56372680	2.49E-04	T	0.065	GRMZM2G099255	Core-2/I-branching beta-1,6-N-acetylglucosaminyltransferase family protein	other
Chr.5_182033002	1.99E-04	C	0.071	GRMZM2G300771	(AGC2, AGC2-1, AtOXI1, OXI1) AGC (cAMP-dependent cGMP-dependent and protein kinase C) kinase family protein	Stress response related
Chr.5_195185908	4.22E-04	C	0.061	GRMZM2G154628	aquaporin protein putative expressed	Stress response related
Chr.7_107926924	1.40E-04	T	0.088	GRMZM2G019183	trehalose synthase putative expressed	Disease resistance related
Chr.7_131442441	1.67E-04	A	0.07	GRMZM2G018044	Eukaryotic aspartyl protease family protein	Enzymes related to hydrolysis
Chr.7_158749101	3.52E-04	T	0.076	GRMZM2G333980	(ATPGIP1, PGIP1) polygalacturonase inhibiting protein	Disease resistance related
Chr.7_165360093	3.62E-04	C	0.071	GRMZM2G111224	inter-alpha-trypsin inhibitor heavy chain-related	other
Chr.7_170031566	2.81E-04	T	0.065	GRMZM2G055607	P-loop containing nucleoside triphosphate hydrolases superfamily	Enzymes related to hydrolysis
Chr.7_174747321	1.78E-04	C	0.079	GRMZM2G434792	Thiamine pyrophosphate dependent pyruvate decarboxylase family	Regulation process
Chr.8_13790236	1.40E-04	A	0.083	GRMZM2G176568	Homeodomain-like transcriptional regulator	Regulation process
Chr.8_13790237	1.40E-04	A	0.083			
Chr.8_117615728	1.83E-04	C	0.085	GRMZM2G163658	(MCM8) minichromosome maintenance 8	other
Chr.9_153449080	3.56E-04	C	0.064	GRMZM2G374986	Homeodomain-like superfamily protein	Regulation process

R^2^ represent proportion of phenotypic variance explained by SNP.

**Table 4:** Comparison of the resistance of the NIL population with the region containing Chr1.S_6433914.

Lines	Umc1292-umc1106	Background recovery rate (%)	Grade of disease
NC14-1	+/+	85.45	5.44 ± 0.19 a
NS15-1	+/+	83.63	5.00 ± 0.33 a
1044	−/−	90.90	6.44 ± 0.19 b
1038	−/−	89.09	6.67 ± 0.00 b
N6	−/−	—	6.33 ± 0.33 b

+ / + : the target region is a homozygous allele of BT-1 (R); −/−: the target region is a homozygous allele of N6 (S).

Small letters represent that the mean difference is significant at the level of p ≤ 0.01(LSD-ANOVA significance test).
