# Supplementary material for: Dissecting the genetic architecture of Fusarium verticillioides seed rot resistance in maize by combining QTL mapping and genome-wide association analysis
Source: Sci Rep. 2017 Apr 19;7:46446. doi: 10.1038/srep46446 (PMC5396065; doi:10.1038/srep46446)
Supplement: Supplementary Tables and Figures [file srep46446-s1.pdf]

Dissecting the genetic architecture of *Fusarium verticillioides* seed rot resistance in maize by combining QTL mapping and genome-wide association analysis

Ming Ju[1a], Zijian Zhou[1, 2a], Cong Mu[1], Xuecai Zhang[1], Jingyang Gao[3], Yakun Liang[3], Jiafa Chen[1], Yabin Wu[1], Xiaopeng Li[1], Shiwei Wang[1], Jingjing Wen[1], Luming Yang[4]*, Jianyu Wu[1]*

[1] College of Agronomy, Henan Agricultural University, Zhengzhou, 450002, China,

[2] Collaborative Innovation Center of Henan Grain Crops, Henan Agricultural University, Zhengzhou 450002, China,

[3]College of Life sciences, Henan Agricultural University, Zhengzhou, 450002, China,

[4]College of Horticulture, Henan Agricultural University, Zhengzhou, 450002, China

[a] These authors contributed equally to this work.

* For correspondence:

Telephone number: 0086-371-63555785; Fax-number: 0086-371-63555790

Email: wujianyu40@126.com; ylmhort@163.com

The email addresses of all co-authors: Ming Ju, jumingzz@163.com;

Zijian Zhou, zhouzijian19900601@163.com; Cong Mu, 897021462@qq.com;

Xuecai Zhang, 46380340@qq.com; Jingyang Gao, 908985207@qq.com;

Yakun Liang, 498822409@qq.com; Jiafa Chen, 25331919@qq.com;

Yabin Wu, wuyabin1215@163.com; Xiaopeng Li, 928642314@qq.com;

Shiwei Wang, 1092646273@qq.com; Jingjing Wen, 283018646@qq.com;

Luming Yang, ylmhort@163.com; Jianyu Wu, wujianyu40@126.com.

Table S1. Information on lines used in GWAS

Lines of GWAS	Structure result	Pedigree information	The grade of disease
2403-JIAO51	Group 1	P Group	5
CML103	Group 1	CIMMYT	5
CML108	Group 1	CIMMYT	2
CML11	Group 1	CIMMYT	4
CML113	Group 1	CIMMYT	3.4
CML115	Group 1	CIMMYT	4.2
CML116	Group 1	CIMMYT	5.6
CML118	Group 1	CIMMYT	4.8
CML121	Group 1	CIMMYT	4.25
CML122	Group 1	CIMMYT	4
CML130	Group 1	CIMMYT	4.6
CML134	Group 1	CIMMYT	3
CML139	Group 1	CIMMYT	3.2
CML140	Group 1	CIMMYT	3
CML141	Group 1	CIMMYT	4.4
CML142	Group 1	CIMMYT	4.4
CML145	Group 1	CIMMYT	3.8
CML147	Group 1	CIMMYT	6.2
CML151	Group 1	CIMMYT	5
CML152	Group 1	CIMMYT	5.4
CML153	Group 1	CIMMYT	4.6
CML154	Group 1	CIMMYT	4
CML155	Group 1	CIMMYT	5.2
CML156	Group 1	CIMMYT	7
CML157	Group 1	CIMMYT	6
CML158	Group 1	CIMMYT	3.8
CML160	Group 1	CIMMYT	7
CML161	Group 1	CIMMYT	3
CML162	Group 1	CIMMYT	3.8
CML163	Group 1	CIMMYT	3
CML164	Group 1	CIMMYT	4.4
CML166	Group 1	CIMMYT	4.75
CML169	Group 1	CIMMYT	0
CML170	Group 1	CIMMYT	3.8
CML171	Group 1	CIMMYT	3.75
CML172	Group 1	CIMMYT	4.8
CML173	Group 1	CIMMYT	0.8
CML174	Group 1	CIMMYT	4
CML175	Group 1	CIMMYT	5
CML179	Group 1	CIMMYT	6.25
CML180	Group 1	CIMMYT	4.25

CML181	Group 1	CIMMYT	2.4
CML182	Group 1	CIMMYT	2
CML184	Group 1	CIMMYT	5
CML185	Group 1	CIMMYT	3.6
CML188	Group 1	CIMMYT	4.6
CML189	Group 1	CIMMYT	6.4
CML190	Group 1	CIMMYT	4.6
CML191	Group 1	CIMMYT	4.4
CML192	Group 1	CIMMYT	2
CML193	Group 1	CIMMYT	1.6
CML194	Group 1	CIMMYT	3.4
CML20	Group 1	CIMMYT	5
CML218	Group 1	CIMMYT	1.2
CML219	Group 1	CIMMYT	0.6
CML220	Group 1	CIMMYT	4.6
CML221	Group 1	CIMMYT	3
CML222	Group 1	CIMMYT	4.4
CML223	Group 1	CIMMYT	2.8
CML225	Group 1	CIMMYT	3
CML226	Group 1	CIMMYT	4.2
CML228	Group 1	CIMMYT	5
CML238	Group 1	CIMMYT	0
CML244	Group 1	CIMMYT	4
CML246	Group 1	CIMMYT	5.4
CML258	Group 1	CIMMYT	5
CML269	Group 1	CIMMYT	3.5
CML27	Group 1	CIMMYT	4.2
CML282	Group 1	CIMMYT	0.4
CML285	Group 1	CIMMYT	2.2
CML288	Group 1	CIMMYT	3.6
CML29	Group 1	CIMMYT	3.2
CML292	Group 1	CIMMYT	2.2
CML295	Group 1	CIMMYT	5
CML300	Group 1	CIMMYT	2.5
CML304	Group 1	CIMMYT	3.8
CML31	Group 1	CIMMYT	4.75
CML312	Group 1	CIMMYT	2
CML323	Group 1	CIMMYT	3.6
CML324	Group 1	CIMMYT	2.25
CML325	Group 1	CIMMYT	2.4
CML326	Group 1	CIMMYT	5.2
CML327	Group 1	CIMMYT	5.6
CML328	Group 1	CIMMYT	2.75
CML332	Group 1	CIMMYT	1.8

CML333	Group 1	CIMMYT	2.2
CML338	Group 1	CIMMYT	3
CML36	Group 1	CIMMYT	5.75
CML367	Group 1	CIMMYT	3.4
CML40	Group 1	CIMMYT	2.4
CML410	Group 1	CIMMYT	3.8
CML411	Group 1	CIMMYT	5.6
CML421	Group 1	CIMMYT	2.4
CML422	Group 1	CIMMYT	4.4
CML424	Group 1	CIMMYT	0
CML428	Group 1	CIMMYT	0
CML435	Group 1	CIMMYT	3.5
CML437	Group 1	CIMMYT	3.4
CML438	Group 1	CIMMYT	3
CML445	Group 1	CIMMYT	2.2
CML469	Group 1	CIMMYT	3.6
CML473	Group 1	CIMMYT	2.2
CML477	Group 1	CIMMYT	2.25
CML478	Group 1	CIMMYT	6
CML480	Group 1	CIMMYT	0
CML481	Group 1	CIMMYT	3.5
CML486	Group 1	CIMMYT	1.6
CML487	Group 1	CIMMYT	2
CML489	Group 1	CIMMYT	1.8
CML491	Group 1	CIMMYT	5
CML492	Group 1	CIMMYT	6
CML493	Group 1	CIMMYT	3.4
CML494	Group 1	CIMMYT	2.8
CML495	Group 1	CIMMYT	2.4
CML496	Group 1	CIMMYT	4.75
CML497	Group 1	CIMMYT	2
CML52	Group 1	CIMMYT	3
CML533	Group 1	CIMMYT	2.4
CML538	Group 1	CIMMYT	1.2
CML80	Group 1	CIMMYT	3
CML84	Group 1	CIMMYT	1.4
CML85	Group 1	CIMMYT	3.2
CML91	Group 1	CIMMYT	2.2
CML92	Group 1	CIMMYT	4.25
CML96	Group 1	CIMMYT	3
CML99	Group 1	CIMMYT	1.8
CML178	Group 3	CIMMYT	6
2369-BT-1	Group 2	Special	1.4
2116-2437	Group 2	TSPT	1.8

W444	Group 2	TSPT	2
12H647-1-TSPT1	Group 2	TSPT	2.4
2115-2434	Group 2	TSPT	2.4
12H567-1-TSPT24	Group 2	TSPT	2.75
12H650-1-TSPT4	Group 2	TSPT	2.8
2123-2344-5	Group 2	TSPT	3
2406-ST255	Group 2	(BT-1×N6)RIL	3
CHANG7-2	Group 2	TSPT	3.4
12H560-1-TSPT10	Group 2	TSPT	3.8
12H556-1-TSPT9	Group 2	TSPT	4
12H568-1-TSPT25	Group 2	TSPT	4
12H574-1-TSPT15	Group 2	TSPT	4
12H652-1-TSPT5	Group 2	TSPT	4
12H562-1-TSPT12	Group 2	TSPT	4.2
12H565-1-TSPT22	Group 2	TSPT	4.2
12H644-1-TSPT18	Group 2	TSPT	4.2
12H561-1-TSPT11	Group 2	TSPT	4.4
12H572-1-TSPT14	Group 2	TSPT	4.4
12Z413-2-TSPT3	Group 2	TSPT	4.4
444	Group 2	TSPT	4.8
2378-N6	Group 2	TSPT	5.6
12H495-1-TSPT19	Group 2	TSPT	6.4
XI502	Group 2	TSPT	6.4
R136	Group 2	(BT-1×N6)RIL	0.4
R166	Group 2	(BT-1×N6)RIL	1.4
R180	Group 2	(BT-1×N6)RIL	1.4
R55	Group 2	(BT-1×N6)RIL	2.4
R205	Group 2	(BT-1×N6)RIL	2.4
R62	Group 2	(BT-1×N6)RIL	2.6
R43	Group 2	(BT-1×N6)RIL	3
R83	Group 2	(BT-1×N6)RIL	3
R132	Group 2	(BT-1×N6)RIL	3
R123	Group 2	(BT-1×N6)RIL	3.2
R201	Group 2	(BT-1×N6)RIL	3.2
R15	Group 2	(BT-1×N6)RIL	3.4
R230	Group 2	(BT-1×N6)RIL	3.4
R235	Group 2	(BT-1×N6)RIL	3.4
R30	Group 2	(BT-1×N6)RIL	3.6
R104	Group 2	(BT-1×N6)RIL	3.6
R137	Group 2	(BT-1×N6)RIL	3.75
R40	Group 2	(BT-1×N6)RIL	4
R87	Group 2	(BT-1×N6)RIL	4
R92	Group 2	(BT-1×N6)RIL	4
R130	Group 2	(BT-1×N6)RIL	4

R165	Group 2	(BT-1×N6)RIL	4
R193	Group 2	(BT-1×N6)RIL	4
R214	Group 2	(BT-1×N6)RIL	4.6
R99	Group 2	(BT-1×N6)RIL	4.8
R22	Group 2	(BT-1×N6)RIL	5
R188	Group 2	(BT-1×N6)RIL	5
12H692-1-P Group2	Group 3	P Group	0.4
12H378-1-Lan6	Group 3	Lancaster	1.4
12H583-1-Reid15	Group 3	Reid	1.8
12H685-1-P Group11	Group 3	P Group	1.8
12H664-1-Reid10	Group 3	Reid	2.6
12H580-1-Reid13	Group 3	Reid	2.8
2193-2013-32	Group 3	Reid	2.8
12H691-1-Lan1	Group 3	Lancaster	3
12HR707-1-P Group4	Group 3	P Group	3
ZHENG58	Group 3	Reid	3
12H605-1-Reid9	Group 3	Reid	3.2
12H678-1-Reid2	Group 3	Reid	3.2
12H690-1-P Group1	Group 3	P Group	3.2
2203-2126-15	Group 3	Reid	3.2
12H640-1-P Group6	Group 3	P Group	3.25
2192-2013-24	Group 3	Reid	3.25
12H602-1-P Group9	Group 3	P Group	3.6
12H623-1-P Group8	Group 3	P Group	3.6
12H642-1-P Group17	Group 3	P Group	4
12H683-1-Reid3	Group 3	Reid	4
12HR711-1-Lan2	Group 3	Lancaster	4
913-Mo17	Group 3	Lancaster	4
12H413-1-P Group13	Group 3	P Group	4.25
12H588-1-Reid18	Group 3	Reid	4.4
12H457-1-P Group14	Group 3	P Group	4.5
2399-72	Group 3	Lancaster	4.5
12H621-1-Reid4	Group 3	Reid	4.6
2204-2126-29	Group 3	Reid	4.6
2397-276	Group 3	Reid	4.75
12H686-1-P Group3	Group 3	P Group	4.8
12H603-1-Reid8	Group 3	Reid	5
12HR714-1-Lv1	Group 3	Luda Red Cob	5
12H667-1-Reid11	Group 3	Reid	5.2
12H641-1-P Group7	Group 3	P Group	5.6
12H698-1-P Group12	mixed group	P Group	4.6
12H577-1-Reid6	mixed group	Reid	4
R91-(BT-N6)RIL106	mixed group	(BT-1×N6)RIL	3.8

Table S2. Physical positions of partial SNPs significantly associated with FSR resistance and the predicted function or homology of candidate genes.

Marker	P-value	Resistant allele	R^2	Gene	Annotation
Chr.1_202169556	0.000413	T	0.066	GRMZM2G123016	expressed protein
Chr.6_140711920	0.000438	G	0.064	GRMZM2G093325	expressed protein
Chr.6_154335211	0.000376	C	0.062	GRMZM2G701206	expressed protein
Chr.7_86275599	0.00026	A	0.077	AC187460.4_FG002	expressed protein
Chr.9_109115056	0.00026	C	0.07	GRMZM2G103900	expressed protein
Chr.9_109115169	0.000238	C	0.07		
Chr.9_109115172	0.000264	C	0.07		
Chr.2_15283638	0.00044	C	0.068	GRMZM2G341111	Non-coding gene
Chr.6_150940467	0.000445	A	0.068	AC208912.2_FG003	Non-coding gene
Chr.8_172132198	0.000406	A	0.07	GRMZM2G417795	Non-coding gene
Chr.9_59770427	0.000354	C	0.067	GRMZM5G846832	Non-coding gene
Chr.7_156316378	0.0000855	A	0.076	NO	NO

R^2 represent proportion of phenotypic variance explained by SNP.

Table S3. Descriptive Statistics and correlations between FSR and FER of 100 maize lines.

Disease Resistance	Mean	Std.Deviation	N	Pearson Correlation	
				FSR	FER
FSR	3.5868	1.2663	100	1	0.148
FER	0.0402	0.06085	100	0.148	1

Table S4. Evaluation standard of *F. verticillioides* seed rot resistance in maize.

Grades of diseases	Symptom Descriptions in one petri dish with 10 seeds
0	SN = 0 and IA = 0%
1	0 < SN ≤ 2 and 0 < IA ≤ 25%
2	SN=2 and IA > 25% or 3 ≤ SN < 7 and 0 < IA < 25%
3	3 ≤ SN < 7 and 25% ≤ IA < 50%, or SN ≥ 7and 0 < IA < 25%
4	3 ≤ SN < 7 and 50% ≤ IA < 75%, or SN ≥ 7 and 25 ≤ IA < 50%
5	3 ≤ SN < 7 and 75% ≤ IA ＜ 100%, or SN ≥ 7 and 50 ≤ IA < 75%
6	3 ≤ SN < 7 and IA=100% , or SN ≥ 7and 75 ≤ IA < 100%
7	SN=10 and IA=100%

Note: SN-symptom diseased seed number; IA- seed incidence area / seed total area

Table S5. Primer used in qRT-PCR.

Gene		Primer (5'-3')
Beta-actin (reference gene)	Sense	CGATTGAGCATGGCATTGTCA
	Anti-sense	CCCACTAGCGTACAACGAA
GRMZM2G0081223	Sense	GCTGTGATGACCGTGGTGTTC
	Anti-sense	CCTGGCTGATGATGCTGACTTG
AC213654.3_FG004	Sense	TACGTCTGCGTCAGCGGACA
	Anti-sense	TGGAAGCGTCATCCTCGGAGTC
GRMZM2G099255	Sense	ACACCCAGTTTATTGCCATTT
	Anti-sense	GCCCCATATCACCTTTTCG

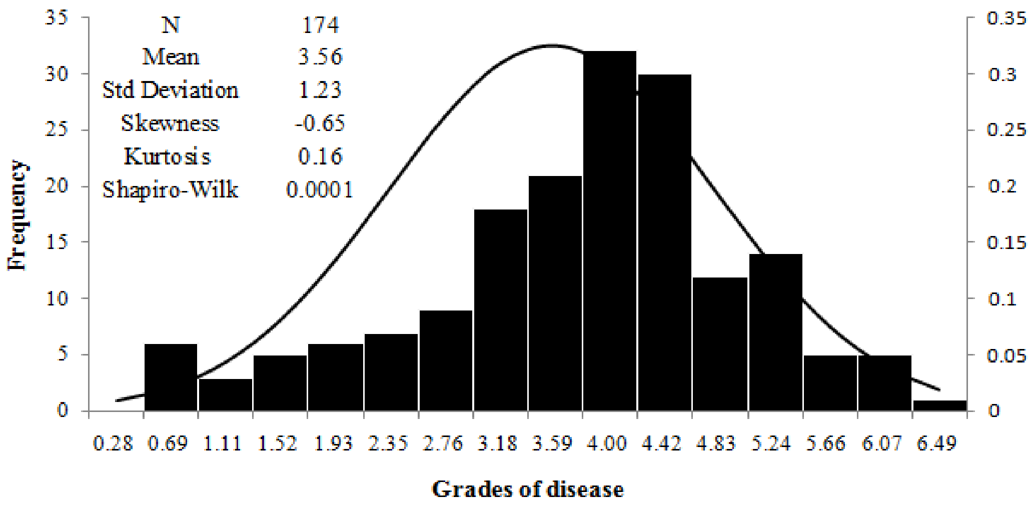

Figure S1. The frequency distribution of grades of disease of 174 individuals in RIL population.

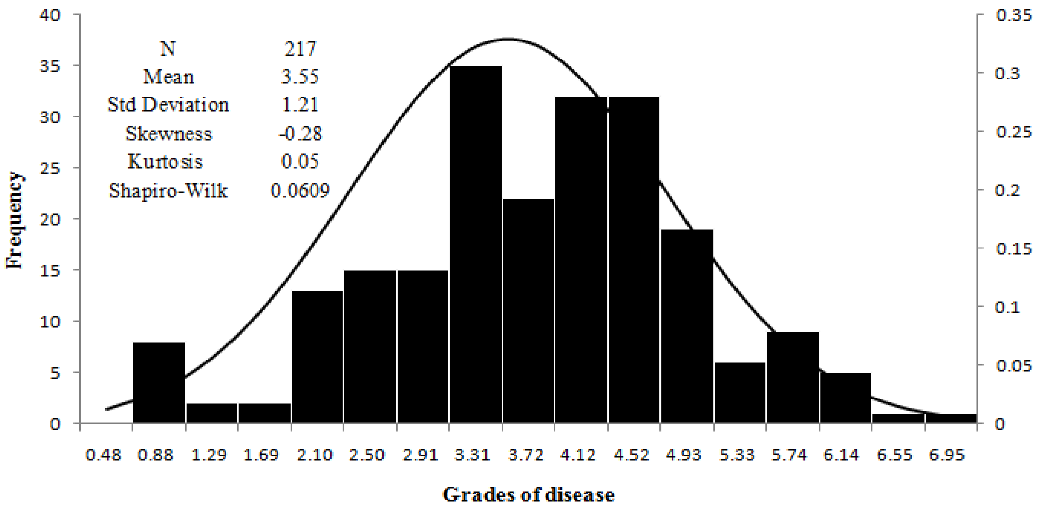

Figure S2. The frequency distribution of grades of disease of 217 maize lines in the association mapping panel.

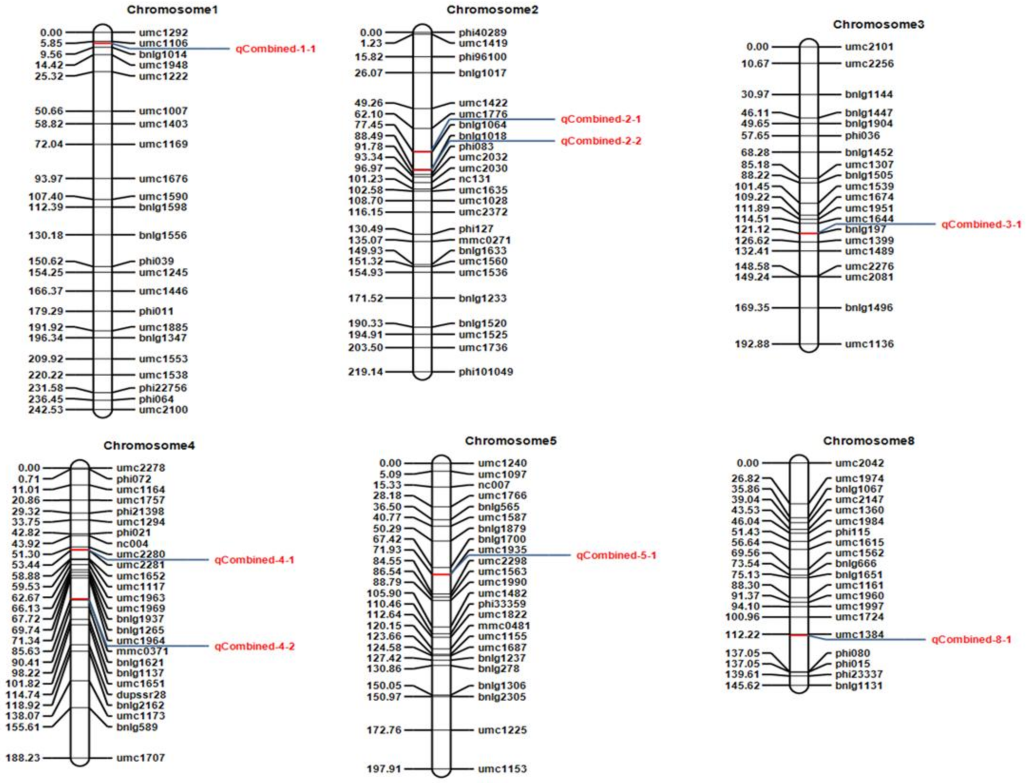

Figure S3. Eight QTLs (PVE: 4.07-12.06%, LOD: 2.57-6.96) associated with the resistance to *F. verticillioides* seed rot on six chromosomes using 174 $F_{9:10}$ mapping population (BT-1×N6) of maize.

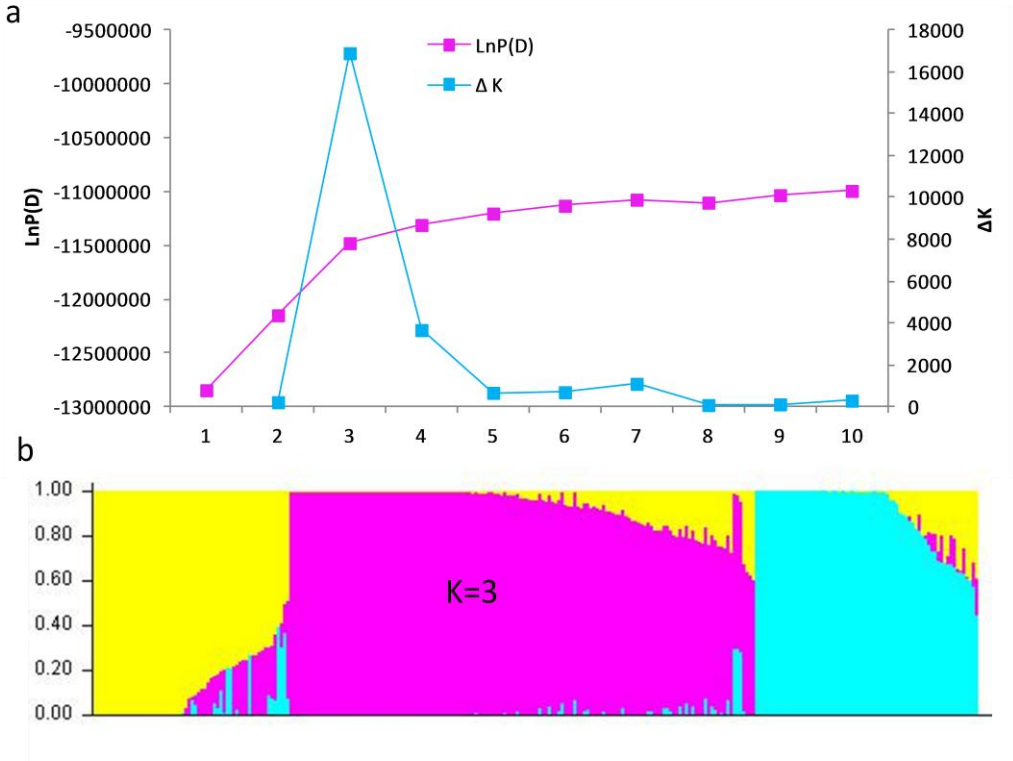

Figure S4. Population structure of 217 maize inbred lines based on 5000 SNP markers: (a) Plot of LnP(D) and an ad hoc statistic ΔK calculated for K ranging from 1 to 10; (b) population structure of the 217 lines when K=3. Each individual is represented by a single vertical line that is partitioned into K colored segments (K=3) in the x-axis, with lengths proportional to the estimated probability membership (y-axis) to each of the K inferred clusters.

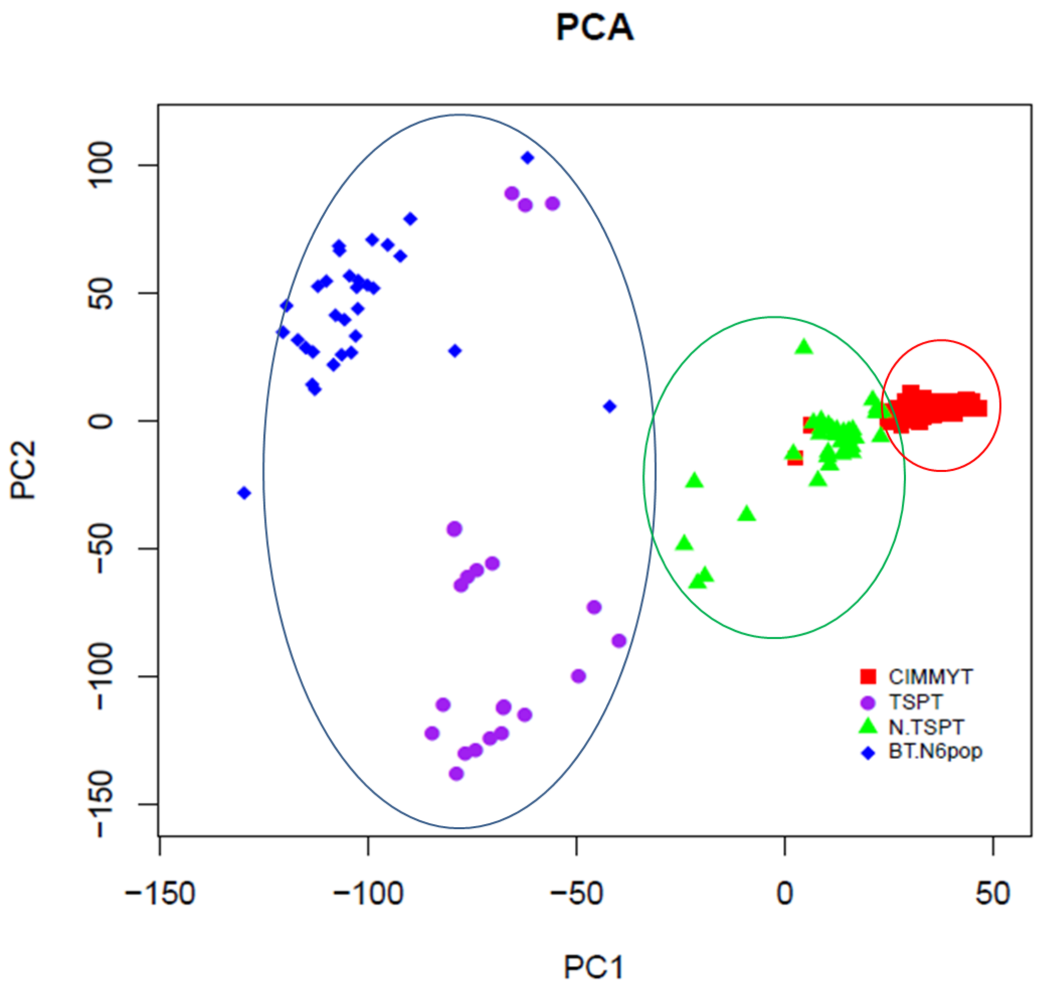

Figure S5. The principal component analysis (PCA) of association mapping panel. CIMMYT represents maize lines collected from CIMMYT; TSPT represents maize lines from Tang Si Ping Tou blood of China materials; N.TSPT represents maize lines from Reid and Lancaster related materials; BT.N6pop represents maize lines from bi-parental of BT-1and N6 RIL population.

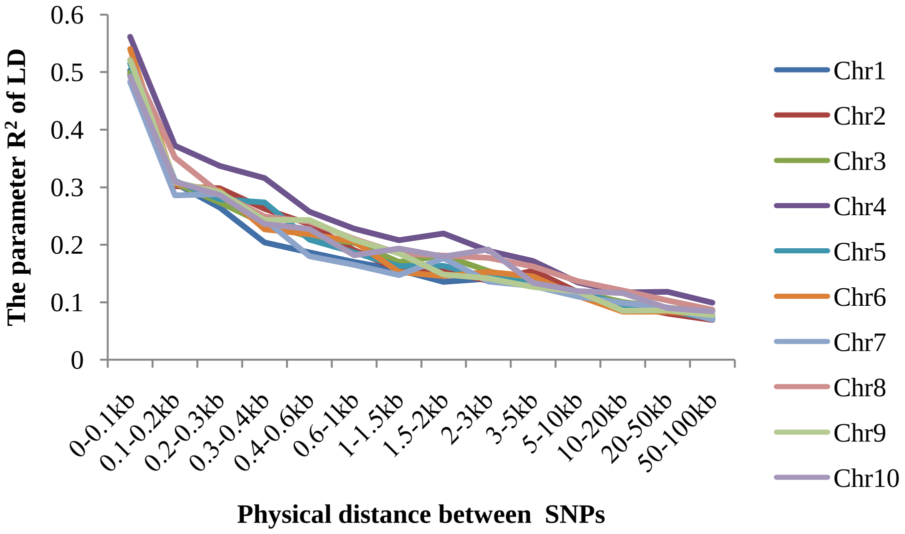

Figure S6. LD decay across 10 chromosomes in maize. The X-axis indicates the physical distance between SNPs within the same chromosome and the Y-axis indicates the parameter R^2 of LD.

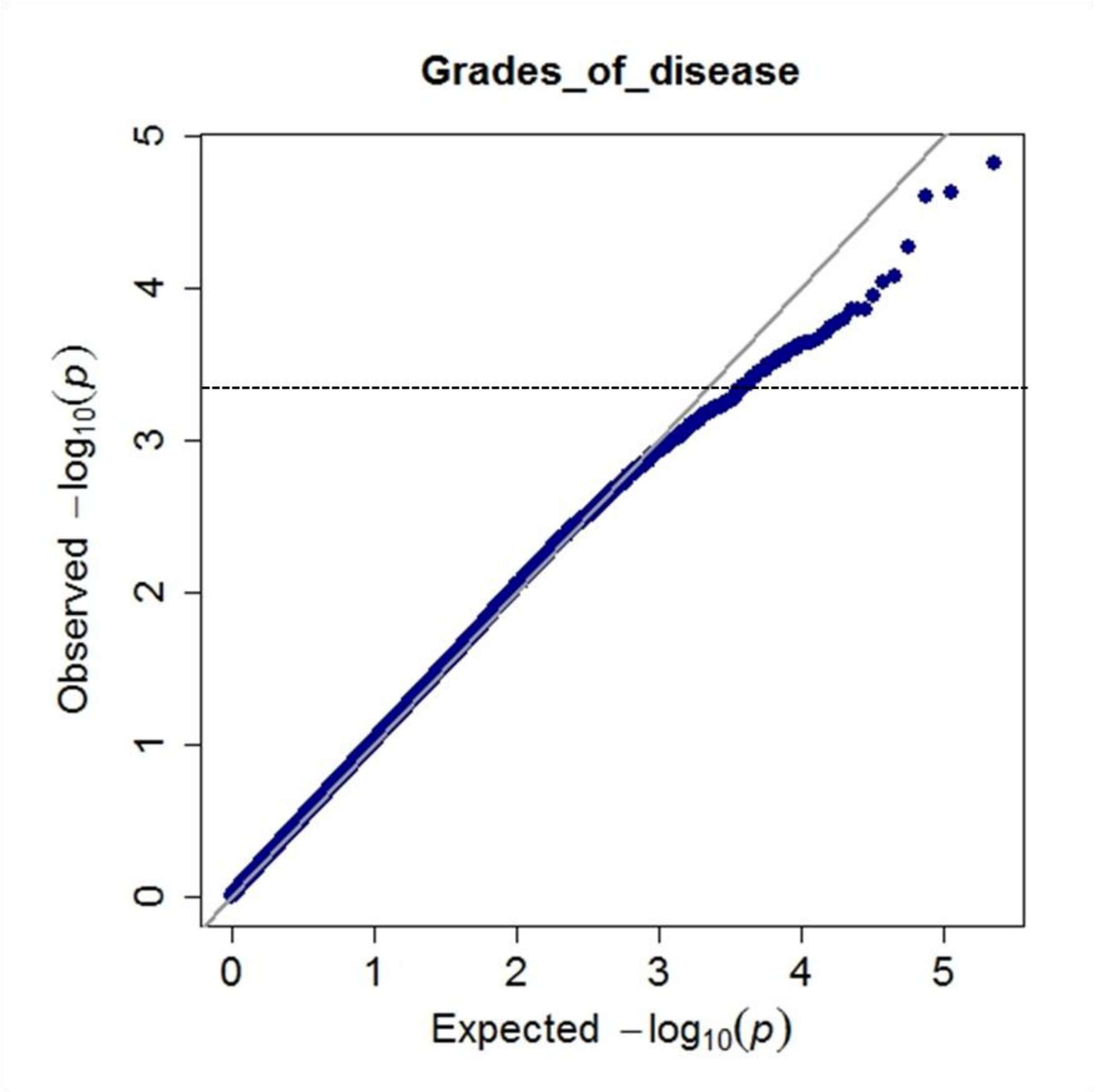

Figure S7. QQ plots from the GWAS results for the grades of disease of *F. verticillioides* seed rot resistance. Plots above the imaginary line show the genome-wide significance with a moderately stringent threshold of 100/number of SNPs.

```
BT-1     MEMTSAMELSLEQIRKEAVDLENISMEEVFAFLKCCKEGLSTEEAQKRVLMFGPNKLEERKESKVLKFLMFMWNPLSWVM     80
N6       MEMTSAMELSLEQIRKEAVDLENISMEEVFAFLKCCKEGLSTEEAQKRVLMFGPNKLEERKESKVLKFLMFMWNPLSWVM     80

BT-1     EMAAVMAIALANGDNRPPDWQDFVGIVVLLVINSTISFVEENNAGSAAEALMANLAPKTKVLRDGQWSEEDAAVLVPGDI    160
N6       EMAAVMAIALANGDNRPPDWQDFVGIVVLLVINSTISFVEENNAGSAAEALMANLAPKTKVLRNGQWSEEDAAVLVPGDI    160

BT-1     ISIKLGDIVPADARLLEGDALKIDQSALTGECLPVTKSPGSSVYSGSTCKQGEIEAVVIATGVHTFFGKAAHLVDSTNQV    240
N6       ISIKLGDIVPADARLLEGDALKIDQSALTGECLPVTKSPGSSVYSGSTCKQGEIEAVVIATGVHTFFGKAAHLVDSTNQV    240

BT-1     GHFQKVLKAIGNFCIGAIAMGVIVEVVVMYAVQHRRYREGIDNLLVLLIGGIPIAMPTVLSVTMAIGSHKLSLQGAITKR    320
N6       GHFQKVLKAIGNFCIGAIAIGVIVEVVVMYAVQHRRYREGIDNLLVLLIGGIPIAMPTVLSVTMAIGSHKLSLQGAITKR    320

BT-1     MTAIEEMAGMDVLCSDKTGTLTLNKLSVDRTLVEIFAAGVTKDDVILFAARASRVENQDAIDAAMVGMLGDPKEARDGIE    400
N6       MTAIEEMAGMDVLCSDKTGTLTLNKLSVDRTLVEIFAAGVTKDDVILFAARASRVENQDAIDAAMVGMLGDPKEARDGIE    400

BT-1     EVHFFPFNPVDKRTALTYIDLADGSWHRVSKGAPEQILALCNCGDDVCNLVHTVIDKYAERGLRSLAVARQQVPEKSKES    480
N6       EVHFFPFNPVDKRTALTYIDLADGSWHRVSKGAPEQILALCNCGDDVCNLVHTVIDKYAERGLRSLAVARQQVPEKSKES    480

BT-1     LGDPWEFVGLLPLLDPPRSDSSDTIKRALDLGVNVKMITGTPRIPRGFFHSRCYVLTCF.....ACIAGDQLAIAKETGR    555
N6       LGDPWEFVGLLPLLDPPRSDSSDTIKRALDLGVNVKMITGTPRIPRGFFHSRCYVLTMAHTTQPACIAGDQLAIAKETGR    560

BT-1     RLGMGTNMYPSSALLGQSKDEATASVPVDDLIEKADGFAGVFPEHKYEIVKRLQEMKHICGMTGDGVNDAPALKKADIGI    635
N6       RLGMGTNMYPSSALLGQSKDEATASVPVDDLIEKADGFAGVFPEHKYEIVKRLQEMKHICGMTGDGVNDAPALKKADIGI    640

BT-1     AVAGATDAARSASDIVLTQEGLSVIISAVLTSRAIFQRMKNYTIYAVSITIRIVLGFLLIALIWKFDFSPFMILVIAILN    715
N6       AVAGATDAARSASDIVLTQEGLSVIISAVLTSRAIFQRMKNYTIYAVSITIRIVLGFLLIALIWKFDFSPFMILVIAILN    720

BT-1     DGTIMTIAKDRVKPSPLPDSWKLNEIFATGVVYGTYMAVMTVVFFWAMRSTDFFSNTFHVRSLRGSTEEMMSALYLQVSI    795
N6       DGTIMTIAKDRVKPSPLPDSWKLNEIFATGVVYGTYMAVMTVVFFWAMRSTDFFSNTFHVRSLRGSTEEMMSALYLQVSI    800

BT-1     ISQALIFVTRSRSWCFAERPGFLLCAAFVIAQIVATLIAVWADFGFAHIRGIGWGWAGVIWLYSVVTFVPLDLFKFAIRY    875
N6       ISQALIFVTRSRSWCFAERPGFLLCAAFVIAQIVATLIAVWADFGFAHIRGIGWGWAGVIWLYSVVTFVPLDLFKFAIRY    880

BT-1     VLAGKAWNNLLQNKTAFTTKKNYGGEERMAQWATTQRSLHGLPVTEPEAGGRRSGSFVELSEVAEQARRRAEFARLREKN    955
N6       VLAGKAWNNLLQNKTAFTTKKNYGGEERMAQWATTQRSLHGLPVTESEAGGRRSGSFVELSEVAEQARRRAEFARLREKN    960

BT-1     TLRGQLESSARLRGVDLNAIKSPFYS                                                          981
N6       TLRGQLESSARLRGVDLNAIKSPFYS                                                          986
```

Figure S8. Sequence alignment of *GRMZM2G0081223* between the parental lines BT-1 and N6.

```
BT-1     ........MPMAPRHLGRSRPAPTPKPFGEHAKWSTTRVSDRAYSILYLLHPPCHCPLRALHRLGRSSRTLWLGLAMSEG     72
N6       MPEWPFVKRRMAPRHLGRSRPAPTPKPFGEHAKWSTTRVSDRAYSILYLLHPPCHCPLRALHRLGRRSRTLWLGLAMSEG     80

BT-1     ARNTRQFFAASTSGSGSSSATYRDGGGGIGVARASLVNTGILDENVLALVFRYLNFDPKALCTVSCVSRRLRAVAERVLW    152
N6       ARNTRQFFAASTSGSGSSSATYRDGGGGIGVARASLVNTGILDENVLALVFRYLNFDPKALCTVSCVSRRLRAVAERVLW    160

BT-1     RELCVSRAPRMVASLAGGGAPPGRVVGGWPALAKLLLFCCGAAAAAVRGHFTGVSRFSKTSGRSFLSRRCRGDLLYVSDP    232
N6       RELCVSRAPRMVASLAGGGAPPGRVVGGWPALAKLLLFCCGAAAAAVRGHFTGVSRFSKTSGRSFLSRRCRGDLLYVSDP    240

BT-1     CEHAVHGADDDVGAYRGVFRGFTRSRTRACLVGRRAPLETRVRCPYCGARVWSMVAAGMAPRSACRRLGAHEGRLQYYVC    312
N6       CEHAVHGADDDVGAYRGVFRGFTRSRRRACLVGRRAPLETRVRCPYCGARVWSMVAAGMAPRSACRRLGAHEGRLQYYVC    320

BT-1     VSGHLHGNCWLARLTSSDADRDADSEDDASTEGGDDGAHVA                                          353
N6       VSGHLHGNCWLARLTSSDADRDADSEDDASTEGGDDGAHVA                                          361
```

Figure S9. Sequence alignment of *AC213654.3_FG004* between the parental lines BT-1 and N6.

```
BT-1    MAPRSRAPSRRPLWFIVLVAFVCVVAIGAYLYTPQHYTACYLVPSEACNSRPPPEPARVYTDDEIAARVIMRDIIRARPV     80
N6      MAPRSRAPSRRPLWFIVLVAFVCVVAIGAYLYTPQHYTACYLVPSEACNSRPPPEPARVYTDDEIAARVIMRDIIRARPV     80

BT-1    QSKNTKIAFMFLTPSLLPFEKLWEKFFMGHEDRYTIYVHASRDIPSHSSPIFAGRDIRSEKVIWGTISMLDAEKRLLAHA    160
N6      QSKNTKIAFMFLTPSLLPFEKLWEKFFMGHEDRYTIYVHASRDIPSHSSPIFAGRDIRSEKVIWGTISMLDAEKRLLAHA    160

BT-1    LQDPENQHFVLLSESCVPLHNFDYIYSYLMETNVSFVDCFDDPGPHGAGRYSEHMLPEIVKKDWRKGAQWFTVKRQHAIL    240
N6      LQDPENQHFVLLSESCVPLHNFDYIYSYLMETNVSFVDCFDDPGPHGAGRYSEHMLPEIVKKDWRKGAQWFTVKRQHAIV    240

BT-1    ILADTLYYGKFKRYCKPGNEWHNCYSDEHYLPTLFNVSVQKLAPLLFSSISQQHFLPR..                        298
N6      ILADTLYYGKFKRYCKPGSEWHNCYSDEHYLPTLFNVSVQKLAPFCSLRSANNIFYREKK                        300
```

Figure S10. Sequence alignment of *GRMZM2G099255* between the parental lines BT-1 and N6.

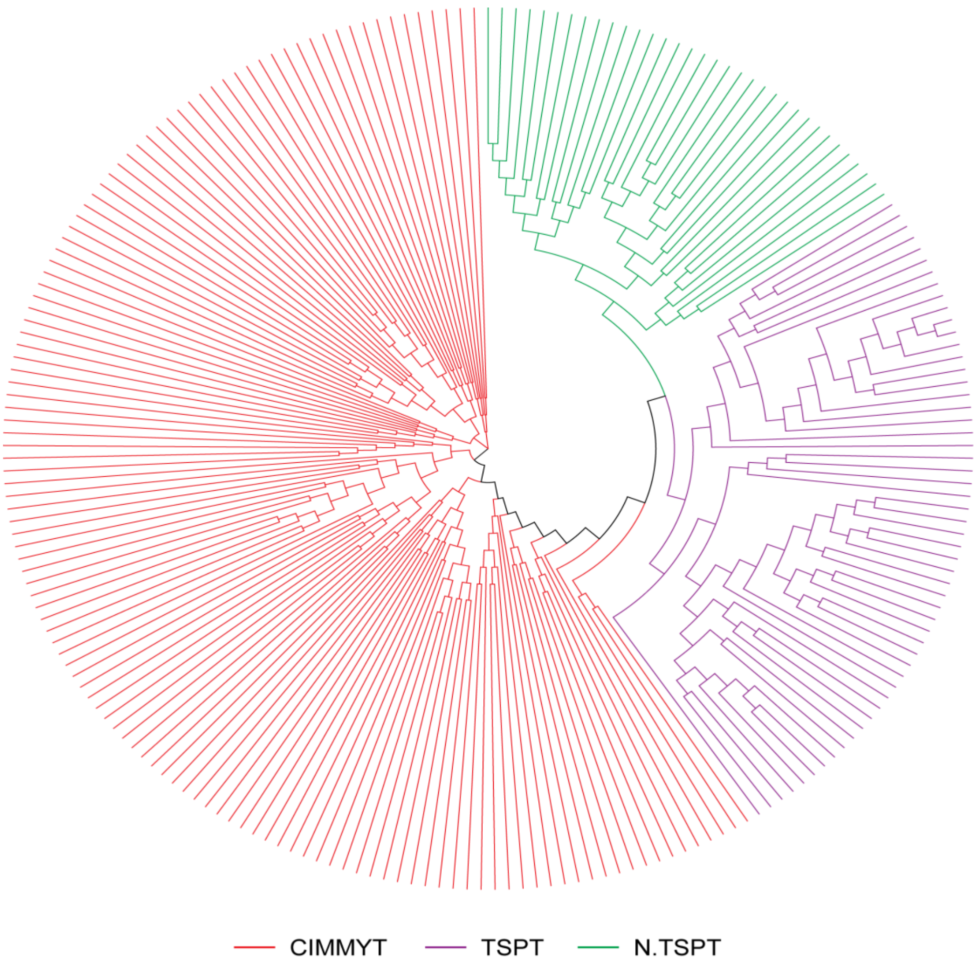

Figure S11. The phylogenetic tree of association panel based on neighbor joining (NJ) method. CIMMYT represents these maize lines collected from CIMMYT; TSPT represents these lines from Tang Si Ping Tou blood of China materials and some material from BT.N6pop; N.TSPT represents these lines from Reid and Lancaster related materials.

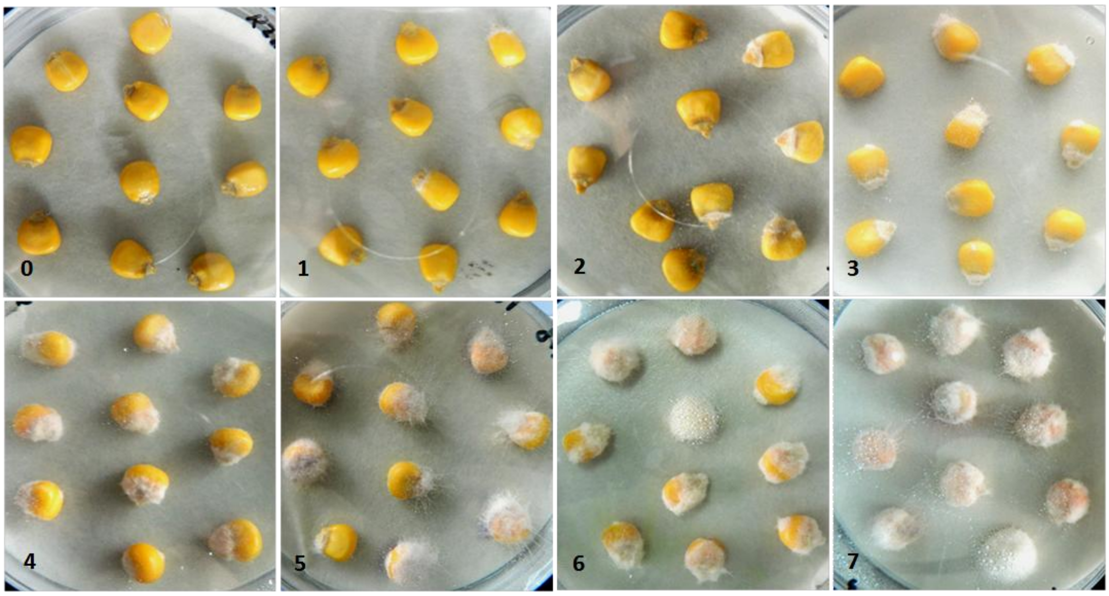

Figure S12. Symptom of maize seeds resistance to *F. verticillioides* in one petri dish. Note: 0-7 represents the grades of diseases, respectively.

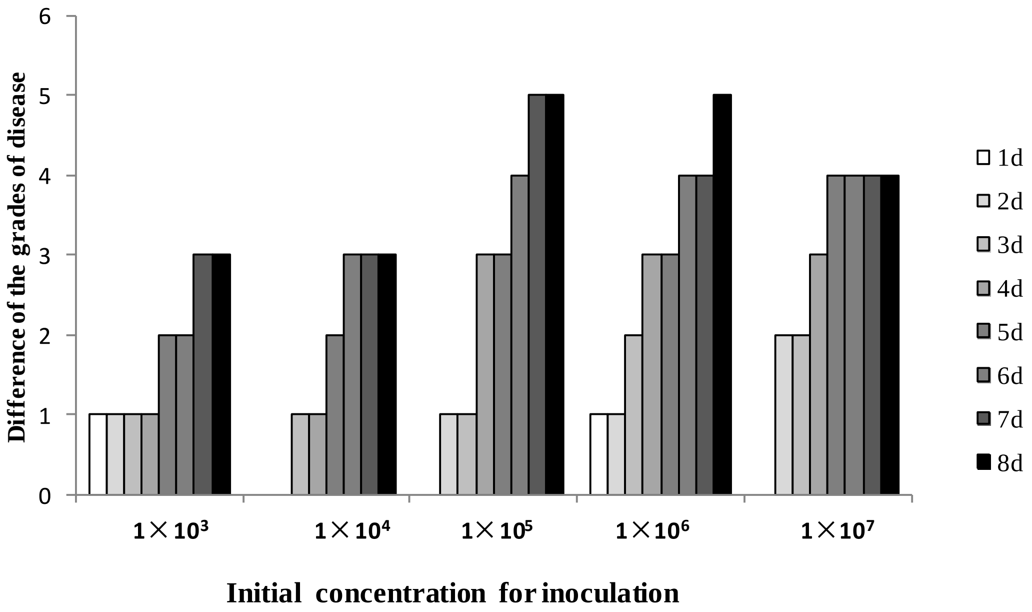

Figure S13. Difference of the grades of disease between BT-1 and N6 under different spore concentration and different period.
